# Supplementary material for: Asynchronous magnetic resonance elastography: Shear wave speed reconstruction using noise correlation of incoherent waves
Source: Magn Reson Med. 2022 Oct 27;89(3):990–1001. doi: 10.1002/mrm.29502 (PMC9792433; doi:10.1002/mrm.29502)
Supplement: Supplementary file 1 — DATA S1 MATLAB code used to generate the numerical simulations of Figure 1 [file MRM-89-990-s002.zip › k-Wave/helpfiles/example_cpp_running_simulations.html]

Running C++ Simulations Example (k-Wave)


# Running C++ Simulations Example

This example demonstrates how to use the C++ versions of `kspaceFirstOrder3D`. Before use, the appropriate C++ codes must be downloaded from http://www.k-wave.org/download.php and placed in the `binaries` folder of the toolbox.

- Open the file in the MATLAB Editor
- Run the file in MATLAB

## Contents

- MATLAB and C++
- Running simulations using the C++ code
- Reloading the output data into MATLAB
- Running the C++ code directly from MATLAB
- Running GPU simulations using the C++ CUDA code

## MATLAB and C++

MATLAB is a very powerful environment for scientific computing. It interfaces with a number of highly-optimised matrix and maths libraries, and can be programmed using relatively simple syntax. User written MATLAB code is also highly portable across operating systems and computer platforms. The portability comes from MATLAB being an interpreted language. This means the code is analysed and executed line by line as the program runs, without needing to be compiled into machine code in advance. In most situations, this means the k-Wave Toolbox works straight out of the (virtual) box on any computer installed with MATLAB.

The downside of MATLAB being an interpreted language is that it can be difficult to optimise user code. Within the main time loop of the k-Wave simulation functions, the code consists primarily of FFTs and element-wise matrix operations like multiplication and addition. Executing these tasks involves a large number of memory operations for relatively few compute operations. For example, to multiply two matrices together element by element, the individual matrix elements are transferred from main memory to cache in blocks, multiplied together using the CPU, and then the results transferred back to main memory. If the matrix elements were already stored in cache, the multiplication would be an order of magnitude faster. However, because the lines of code in MATLAB are executed independently, there is no way in MATLAB to fuse together multiple operations to maximise the temporal and spatial locality of the data.

For simulations using small grid sizes, the compute times are short enough that the drawbacks of MATLAB being an interpreted language are not a concern. However, for simulations using large grid sizes (for example, 512 by 512 by 512), the compute times in MATLAB can stretch into tens of hours. To reduce these compute times, k-Wave also includes optimised versions of `kspaceFirstOrder2D`, `kspaceFirstOrderAS`, and `kspaceFirstOrder3D` written in C++ . The code for desktop computers and servers is called `kspaceFirstOrder-OMP` (the same binary is used for simulations in 2D, 3D, and axisymmetric coordinates). This is optimised for shared memory computer architectures, including machines with multiple CPUs based on a non-uniform memory access (NUMA) design. The code for NVIDIA graphics processing units (GPUs) is called `kspaceFirstOrder-CUDA` (the same binary is used for simulations in 2D and 3D - axisymmetric simulations are not currently supported).

## Running simulations using the C++ code

The C++ code has been designed as a standalone application that can be used completely independently of MATLAB. This is important when using computers and servers that do not have MATLAB support. For this reason, simulation data must be transferred between MATLAB and the C++ code using external input and output files. These files are stored using the Hierarchical Data Format HDF5. The input file defines the properties of the grid, medium, source, and sensor in the same way as the input files passed to the MATLAB code. The output file contains the recorded simulation data along with performance statistics like the compute time and memory usage. A more detailed discussion of the file format and properties is given in the k-Wave manual.

Although the required input file may be created using C++ or any programming language that supports HDF5, for most users it will be easiest to use the MATLAB functions `kspaceFirstOrder2D`, `kspaceFirstOrderAS`, and `kspaceFirstOrder3D` to create the input matrices and save them to disk in the required format (note, this requires MATLAB 2011a or later). The HDF5 input file is automatically generated by adding the flag `'SaveToDisk'` and a filename (or pathname and filename) to the optional input arguments as shown below. When this flag is given, the MATLAB code runs the preprocessing steps, saves the input parameters to disk, and then aborts without running the actual simulation (set `example_number = 1` within the example m-file).

```
% save input files to disk
filename = 'example_input.h5';
kspaceFirstOrder3D(kgrid, medium, source, sensor, 'SaveToDisk', filename);
```

After saving the input data, the compiled C++ code `kspaceFirstOrder-OMP` can be called from a terminal window or the command prompt. The code requires two command line parameters to specify the input and output files, in addition to a number of optional parameters and flags (a full list is given in the k-Wave manual). For example, assuming the input file is located in a root folder called `data`, in Linux, the C++ code can be run from a terminal window using the syntax

```
./kspaceFirstOrder-OMP -i /data/example_input.h5 -o /data/example_output.h5
```

Similarly, in Windows, the C++ code can be run from the command prompt using the syntax

```
kspaceFirstOrder-OMP.exe -i C:\data\example_input.h5 -o C:\data\example_output.h5
```

By default, the time varying pressure at the grid points specified by the sensor mask are recorded during the simulation. When using the MATLAB code, it is possible to control the acoustic variables that are recorded during the simulation by setting the value of `sensor.record`. For the C++ code, the same behaviour is achieved using additional command line parameters (a full list is given in the k-Wave manual). For example, the final pressure field and the particle velocity can be recorded using the syntax

```
./kspaceFirstOrder-OMP -i /data/example_input.h5 -o /data/example_output.h5 --p_final --p_max
```

As the code runs, status updates and computational parameters are printed to the command line. The verbosity of the outputs can be controlled using the `--verbose` command line argument.

```
+---------------------------------------------------------------+ 
|                   kspaceFirstOrder-OMP v1.3                   | 
+---------------------------------------------------------------+ 
| Reading simulation configuration:                        Done | 
| Number of CPU threads:                                     16 | 
| Processor name:     Intel(R) Xeon(R) CPU E5-1660 v3 @ 3.00GHz | 
+---------------------------------------------------------------+ 
|                      Simulation details                       | 
+---------------------------------------------------------------+ 
| Domain dimensions:                             256 x 128 x 64 | 
| Medium type:                                               3D | 
| Simulation time steps:                                   1200 | 
+---------------------------------------------------------------+ 
|                        Initialization                         | 
+---------------------------------------------------------------+ 
| Memory allocation:                                       Done | 
| Data loading:                                            Done | 
| Elapsed time:                                           0.04s | 
+---------------------------------------------------------------+ 
| FFT plans creation:                                      Done | 
| Pre-processing phase:                                    Done | 
| Elapsed time:                                           0.02s | 
+---------------------------------------------------------------+ 
|                    Computational resources                    | 
+---------------------------------------------------------------+ 
| Current host memory in use:                             212MB | 
| Expected output file size:                                8MB | 
+---------------------------------------------------------------+ 
|                          Simulation                           | 
+----------+----------------+--------------+--------------------+ 
| Progress |  Elapsed time  |  Time to go  |  Est. finish time  | 
+----------+----------------+--------------+--------------------+ 
|     0%   |        0.125s  |     74.898s  |  01/08/19 09:05:06 | 
|     5%   |        2.268s  |     41.620s  |  01/08/19 09:04:35 | 
|    10%   |        4.130s  |     36.494s  |  01/08/19 09:04:32 | 
|    15%   |        5.986s  |     33.485s  |  01/08/19 09:04:31 | 
|    20%   |        7.921s  |     31.356s  |  01/08/19 09:04:31 | 
|    25%   |        9.998s  |     29.728s  |  01/08/19 09:04:31 | 
|    30%   |       12.585s  |     29.134s  |  01/08/19 09:04:34 | 
|    35%   |       14.535s  |     26.797s  |  01/08/19 09:04:33 | 
|    40%   |       19.321s  |     28.781s  |  01/08/19 09:04:39 | 
|    45%   |       22.935s  |     27.843s  |  01/08/19 09:04:42 | 
|    50%   |       25.300s  |     25.132s  |  01/08/19 09:04:42 | 
|    55%   |       29.590s  |     24.048s  |  01/08/19 09:04:46 | 
|    60%   |       31.868s  |     21.098s  |  01/08/19 09:04:45 | 
|    65%   |       34.129s  |     18.243s  |  01/08/19 09:04:44 | 
|    70%   |       36.733s  |     15.618s  |  01/08/19 09:04:44 | 
|    75%   |       38.785s  |     12.814s  |  01/08/19 09:04:43 | 
|    80%   |       41.020s  |     10.148s  |  01/08/19 09:04:43 | 
|    85%   |       43.007s  |      7.491s  |  01/08/19 09:04:42 | 
|    90%   |       45.128s  |      4.922s  |  01/08/19 09:04:41 | 
|    95%   |       47.232s  |      2.399s  |  01/08/19 09:04:41 | 
+----------+----------------+--------------+--------------------+ 
| Elapsed time:                                          49.65s | 
+---------------------------------------------------------------+ 
| Sampled data post-processing:                            Done | 
| Elapsed time:                                           0.06s | 
+---------------------------------------------------------------+ 
|                            Summary                            | 
+---------------------------------------------------------------+ 
| Peak memory in use:                                     215MB | 
+---------------------------------------------------------------+ 
| Total execution time:                                  49.84s | 
+---------------------------------------------------------------+ 
|                       End of computation                      | 
+---------------------------------------------------------------+
```

## Reloading the output data into MATLAB

After the C++ code has executed, the output files can be reloaded into MATLAB using the function `h5read` (set `example_number = 2` within the example m-file, ensuring that the path to the output file is correct). The variables stored in the HDF5 output file have the identical names to the MATLAB code. For example, for a simulation run with the `--p_final` and `--p_max` flags, equivalent to setting `sensor.record = {'p_final', 'p_max'}`, the output data can be loaded into MATLAB as shown below.

```
% load output data from the C++ simulation
sensor_data.p_final = h5read('example_output.h5', '/p_final');
sensor_data.p_max   = h5read('example_output.h5', '/p_max');
```

For the simulation in the example file, the source is a square piston driven by a continuous wave, and the medium has a heterogeneous inclusion in the beam path. The expected output from the simulation is shown below.

Note, not all simulation options are currently supported by the C++ code. The sensor mask must be given as a binary matrix or a list of cuboid corners (Cartesian sensor masks are not supported), and all display parameters are ignored as the C++ code does not have a graphical output. Moreover, if using a Transducer as the sensor (e.g., as used in the Simulating B-mode Ultrasound Images Example), the returned sensor data will be different to the MATLAB code. This is because the C++ code returns the time series recorded at each grid point, rather than averaged across each physical sensor element. The C++ sensor data can be converted to the MATLAB format using the `combine_sensor_data` method of the `kWaveTransducer` class (note this is automatically called if using `kspaceFirstOrder3DC` as described below).

```
sensor_data = transducer.combine_sensor_data(sensor_data)
```

## Running the C++ code directly from MATLAB

In addition to calling the C++ code from a terminal or command window, it is also possible to run the C++ code directly from MATLAB. To run the code blindly, calls to `kspaceFirstOrder3D` can be directly substituted with calls to `kspaceFirstOrder3DC` (similarly for 2D and AS simulations) without any other changes (set `example_number = 3` within the example m-file). This function automatically adds the `'SaveToDisk'` flag, calls `kspaceFirstOrder3D` to create the input variables, calls the C++ code using the `computer` command, reloads the output variables from disk using `h5read`, then deletes the input and output files. This is useful when running MATLAB interactively. The disadvantage of running the C++ code from within MATLAB is the additional memory footprint of having many variables allocated in memory twice, as well as the overhead of running MATLAB.

## Running GPU simulations using the C++ CUDA code

In addition to the CPU code, k-Wave also includes an optimised C++/CUDA code for running simulations on graphics processing units (GPUs). The code is called `kspaceFirstOrder-CUDA` and is used in the identical way to `kspaceFirstOrder-OMP`. The code requires a CUDA-enabled NVIDIA GPU with a minimum compute capability of 1.3 and a recent NVIDIA driver. To run the code directly from MATLAB, calls to `kspaceFirstOrder2D` and `kspaceFirstOrder3D` can be directly substituted with calls to `kspaceFirstOrder2DG` and `kspaceFirstOrder3DG` (set `example_number = 4` within the example m-file).
